# Sex differences in perceived expectations of the outcome of total hip and knee arthroplasties and their fulfillment: an observational cohort study

**DOI:** 10.1007/s00296-022-05240-y

**Published:** 2022-11-28

**Authors:** Daisy A. J. M. Latijnhouwers, Thea P. M. Vliet Vlieland, Willem Jan Marijnissen, Pieter-Jan Damen, Rob G. H. H. Nelissen, Maaike G. J. Gademan, The Longitudinal Leiden Orthopaedics Outcomes of Osteoarthritis Study (LOAS) Group, H. M. J. van der Linden, B. L. Kaptein, S. H. M. Verdegaal, H. H. Kaptijn, S. B. W. Vehmeijer, R. Onstenk

**Affiliations:** 1 Department of Orthopaedics, Leiden University Medical Center, Leiden, The Netherlands; 2 Department of Orthopedics, Rehabilitation and Physical Therapy, Leiden University Medical Center, Leiden, The Netherlands; 3 Department of Orthopaedics, Albert Schweitzer Hospital, Dordrecht, The Netherlands; 4 Department of Orthopaedics, Dijklander Hospital, Purmerend, The Netherlands; 5 Department of Clinical Epidemiology, Leiden University Medical Center, Leiden, The Netherlands

**Keywords:** Arthroplasty, Replacement, Hip, Knee, Sex, Expectations, Perception

## Abstract

The influence of sex on preoperative expectations and their fulfillment following total hip and knee arthroplasty (THA/TKA) remains unexplored. We investigated differences between men and women in perceived preoperative expectations on the outcome of THA/TKA and their fulfillment 1 year postoperatively. We performed a cohort study. Expectations were collected preoperatively and 1 year postoperatively using the Hospital for Special Surgery Hip/Knee Replacement Expectations Surveys (HSS-HRES/KRES; not applicable = 0, applicable: back to normal = 1, much = 2/moderate = 3/slight improvement = 4). Fulfillment of expectations was calculated by subtracting preoperative from postoperative scores (score < 0:unfulfilled; score ≥ 0:fulfilled). We included patients with “applicable” expectations. Chi-square and ordinal regression were used to compare expectations and fulfillment regarding sex. 2333 THA (62% women) and 2398 TKA (65% women) patients were included. 77% of THA and 76% of TKA patients completed the HSS-HRES/HSS-KRES both preoperatively and 1 year postoperatively. Men more often perceived items as “applicable”, with differences in 9/20 (HSS-HRES) and 9/19 (HSS-KRES) preoperative items and, respectively, 12/20 (HSS-HRES) and 10/19 (HSS-KRES) postoperative items. The largest differences (> 10%) were found in sexual activity and working ability. 16/20 (HSS-HRES) and 14/19 (HSS-KRES) items showed an increased probability of having higher preoperative expectations of ≥ 10%, in favor of men. In all items, 60% of the respondents indicated that their expectation was fulfilled. Differences were observed in 16/20 (HSS-HRES) and 6/19 (HSS-KRES) items in favor of men. Sex differences were present in expectations and fulfillment, with higher applicability of items, preoperative expectations and fulfillment in men, especially on items related to functional activities.

*Trial registration:* Trial-ID NTR3348.

**Supplementary Information:**

The online version contains supplementary material available at 10.1007/s00296-022-05240-y.

## Introduction

Studies have shown that women more often suffer from hip or knee osteoarthritis (OA), and in general are known to experience more functional limitations and pain before total hip or knee arthroplasty (THA/TKA) compared to men [1, 2]. While previous literature has reported that women show similar or greater postoperative improvements in function and pain than men, women do not seem to achieve the same postoperative levels in function and pain [3–6]. Therefore, Karlson et al. [7] suggested that women might receive THA/TKA at a more advanced stage of OA.

Several explanations for sex differences in preoperative disease state and arthroplasty utilization have been explored. Possible causes could be gender bias in informed decision making, or differences in referral and recommendation of arthroplasty surgery by sex [8, 9]. Additionally, differences in perceptions and expectations between men and women could also be an underlying explanation for the differences found between men and women before and after arthroplasty surgery [7, 10].

Studies investigating to what extent sex affects preoperative expectations and their fulfillment after hip and knee arthroplasty surgery are scarce. Previous literature suggests that female sex is a predictor of lower preoperative expectations on treatment outcomes [7, 11, 12]. Although studies on hip and knee arthroplasties did not investigate differences in specific expectation domains or items between men and women, one study on shoulder arthroplasty did [13]. They found that men were more focused on participation in sports and maintaining their employment, while women valued the ability to independently perform household chores and daily routine most. Studies in joint arthroplasty surgery population show that men mainly focus on activities achievable after surgery, while women are more concerned about the inability to perform basic functional activities after surgery [7, 11–14].

With regard to the fulfillment of the preoperative expectations, Harmsen et al.(Include reference of Harmsen et al. number: [15]) and Tilbury et al. [16] showed that almost 50% of THA/TKA patients had unfulfilled expectations of certain functional outcomes, but did not go into detail whether sex affected these expectations. Two other studies indicate that male sex is associated with higher fulfillment of expectations [17, 18]. However, none of the studies specified the areas in which preoperative expectations or fulfillment differ between men and women. When orthopedic surgeons want to target expectations and fulfillment in expectations, focusing on differences as a result of sex can be helpful in educating and managing expectations [19].

Since available evidence on sex differences on expectations and fulfillment after THA/TKA is scarce, we performed a large observational cohort study. The aim of this study was to investigate differences between men and women in perception of preoperative expectations on outcome of THA/TKA and their fulfillment 1 year postoperatively. Insight into the perception of men and women could improve the preoperative expectation management and shared decision-making process, thereby improving clinical outcomes.

## Materials and methods

### Study design

Consecutive patients undergoing primary THA/TKA as a result of OA were included (June 2012-December 2018) from the ongoing multicenter cohort [Longitudinal Leiden Orthopaedics and Outcomes of OsteoArthritis Study (LOAS), Trial-ID NTR3348] [20]. Patients with an indication for THA/TKA in seven hospitals were included (Leiden University Medical Center; Alrijne Hospital Leiden/Leiderdorp; Waterland Hospital; Albert Schweitzer Hospital; LangeLand Hospital; Groene Hart Hospital; Reinier de Graaf Hospital).

### Patient recruitment

Patients eligible for the LOAS were 18 years or older and able to read and complete the Dutch questionnaires. Patients were excluded if the index procedure was revision hip/knee arthroplasty or if the patient had a diagnosis other than OA. Patients receiving a revision of their primary index arthroplasty within the first year were not excluded. After providing informed consent, questionnaires were filled out preoperatively and 1 year after surgery. The current study population consisted of patients who answered at least one item of the validated Dutch versions of the Hospital for Special Surgery Hip or Knee Replacement Expectations Survey (HSS-HRES/HSS-KRES) prior to surgery. Missing information in any of the other questionnaires was not an exclusion criterion.

### Sociodemographic and clinical characteristics

The following preoperative sociodemographic characteristics were extracted from medical files: sex, age (years), body mass index (BMI) and current smoking status (yes/no). Prior to surgery, information regarding living situation (living alone (yes/no)) and work status (paid work (yes/no); among patients below the Dutch retirement age of 67 years old) was collected using questionnaires. To describe clinical characteristics of the patients, patient-reported outcome measures (PROMs) were gathered preoperatively. Validated Dutch versions of the Hip disability or Knee injury and Osteoarthritis Outcome Score (HOOS/KOOS) were used to assess OA-related problems on five domains: pain, symptoms, function in daily living, function in sports and recreation, and hip or knee-related quality of life (QoL) (score of 100 representing the best possible outcome, 0 meaning the worst outcome; with minimal clinical important differences ranging between 6 and 9) [21, 22]. The Physical and Mental Component Summary of the Short Form-12 (PCS-12 and MCS-12) were used to collect information on the physical and mental health status of the patient (ranging from 0 to 100, with higher scores representing better health) [23]. Preoperative information on comorbidity was collected using the comorbidity questionnaire of the Dutch Central Office of Statistics (CBS) [24]. Both musculoskeletal and non-musculoskeletal comorbidities were dichotomized (yes/no).

### Expectations

Expectations were measured preoperatively and 1 year postoperatively using validated Dutch versions of HSS-HRES and HSS-KRES. Validated Dutch versions of the questionnaires are included in Supplement A [25]. The HSS-HRES contains 20 items and the HSS-KRES 19 items, obtaining patients’ expectations regarding postoperative pain, function, activities and psychological well-being (item is expected: to get back to normal = 1, show much = 2/moderate = 3/slight improvement = 4, or not applicable = 0). One year postoperative, patients received the HSS-HRES/KRES and were asked to report the perceived actual outcome of the items from the preoperative questionnaire (went back to normal = 1, much = 2/moderate = 3/slight improvement = 4, and not applicable = 0). Both not applicable (NA) and missing values were coded as 0. An item was “applicable” if a patient reported ‘back to normal’ or ‘much/moderate/slight improvement’. Only patients that reported an item as “applicable” were included in the analysis to compare expectations. Fulfillment of expectations was determined 1 year postoperative based on the methods used in the study of Tilbury et al. [16]: subtracting the preoperative score from the postoperative score (score < 0: unfulfilled; score ≥ 0: fulfilled; patients with exceeded expectations were also categorized as ‘fulfilled’). When a patient answered "not applicable"(NA) or did not answer an item in either the preoperative or postoperative questionnaire or both, a fulfillment of expectation scorewas not calculated for that item.

Additionally, preoperative scores were transformed to a ‘total expectation score’, ranging from 0 to 76, which was recoded to a 0–100 scale (lowest to highest expectations, respectively). To calculate a total score, ≤ 2 items were allowed to be NA/missing. If more than 2 items were NA/missing, we did not calculate a total score for that patient.

### Statistics

All analyses were stratified by joint (hip/knee). Descriptive statistics were used for the patients’ preoperative characteristics. To assess the presence of potential bias due to dropout, baseline characteristics of patients with and without preoperative expectations were compared by the independent Student’s *T* test (if continuous) or Chi-square test (if categorical) (Supplementary table 1-A). The same tests were performed to identify possible differences in patient characteristics between men and women. After calculating postoperative fulfillment of expectations for each item, frequencies were reported for both THA and TKA patients. “Applicability” of items and postoperative fulfillment of expectations was compared by Chi-square tests to study the similarity of proportions between men and women. Preoperative expectations were compared using ordinal regression, presented as odds ratios (OR) with corresponding 95% confidence intervals (CI; CI lower limit; upper limit) with sex as the determinant and the different items on the HSS-HRES or HSS-KRES as outcome. To our knowledge variables causing both sex and affecting expectations are non-existent. Therefore, in case of our research question, all adjustments would lead to non-preferable adjustments within the causal pathway (Supplement B). As such, analyses performed in this study were not adjusted. Currently, there are no proportions defined for the HSS-HRES and HSS-KRES to discriminate differences between populations. However, to be able to indicate differences between men and women in this study, apart from statistical testing, we reported all differences of ≥ 5% and ≥ 10% between men and women for all items among applicability and fulfillment of expectations to provide additional guidance while reading the most important results. Additionally, we defined OR ≥ 1.1 (indicating a difference in probability of ≥ 10% between men and women) to indicate larger differences in preoperative expectations between men and women. OR > 1 indicated lower preoperative expectations among women, as the highest score represents the lowest value in the HSS-HRES/KRES. Analyses were performed using SPSS version 25.0 (Chicago, IL).

## Results

### Response

**Fig. 1:**
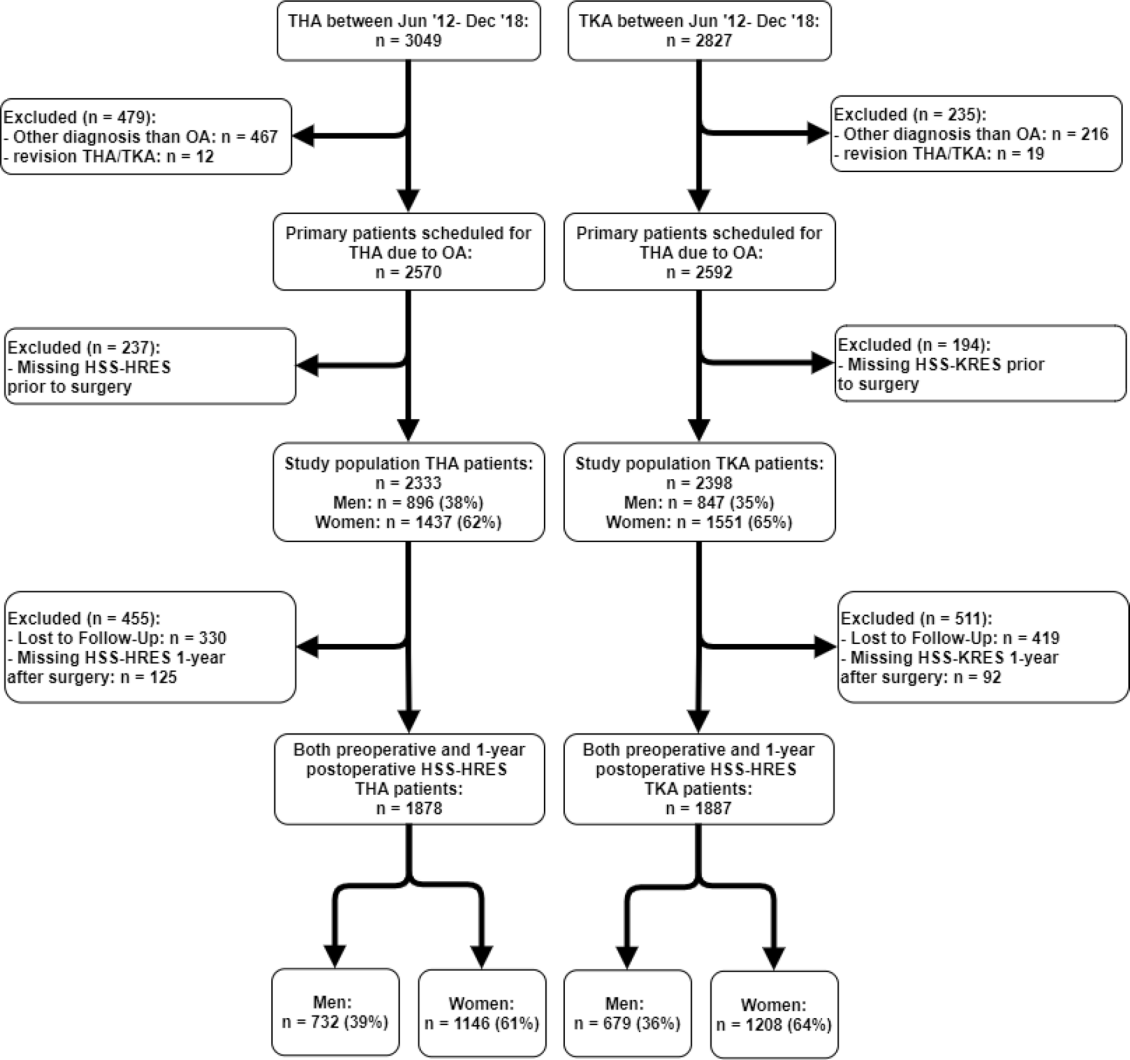
Flowchart of patient selection. Legend: *THA* total hip arthroplasty, *TKA* total knee arthroplasty, *OA* osteoarthritis, *HSS-HRES* Hospital for Special Surgery Hip Replacement Expectations Survey, *HSS-KRES* Hospital for Special Surgery Knee Replacement Expectations Survey

Of the 2570 THA and 2592 TKA patients who were eligible for participation in the study period, 2333 THA (91%) and 2398 TKA (93%) patients filled in the HSS-HRES/KRES questionnaire before surgery and were included. 1878 THA (73%) and 1887 TKA (73%) patients completed both the preoperative and 1 year postoperative questionnaires and were included in the analysis of fulfillment of expectations (Fig. 1). Both the THA and TKA study populations were on average slightly younger, with more comorbidities and better mental health compared to patients without preoperative HSS-HRES/KRES. Additionally, the THA study population was more likely to be employed (Supplementary Table 1-A). We did not find any clinically relevant differences between the populations with HSS-HRES/KRES measurements at both time points compared to the population with only a preoperative HSS-HRES/KRES measurement (Supplementary Table 1-B).

The characteristics of the study population stratified by sex are shown in Table 1. Both the THA and TKA groups have a higher proportion of women (62% of THA and 65% of TKA group). Women were slightly older, suffered more often from comorbidities, reported significantly, but not clinically relevant more OA-related problems (based on lower scores on the HOOS/KOOS subscales), more often lived alone, and were less often employed compared to men. Additionally, women in the TKA population had higher BMI scores than men (30 (5) versus 29 (5)).

**Table 1:** Preoperative characteristics of patients

	THA		TKA	
	Men (*n* = 896)	Women (*n* = 1437)	Men (*n* = 847)	Women (*n* = 1551)
Age, mean (SD) (years)	68 (9)	69 (9)	67 (8)	68 (9)
BMI, mean (SD)	28 (4)	27 (5)	29 (5)	30 (5)
Smoking, yes, *n* (%)	58 (7)	78 (5)	53 (6)	78 (5)
Missing	201 (22)	345 (24)	188 (22)	384 (25)
Comorbidities, *n* (%)				
Non-musculoskeletal	294 (33)	395 (28)	353 (42)	533 (34)
Musculoskeletal	107 (12)	183 (13)	79 (9)	135 (9)
Both	295 (33)	569 (40)	208 (25)	564 (36)
None	81 (9)	86 (6)	114 (14)	131 (8)
Missing	119 (13)	204 (14)	93 (11)	188 (12)
Work status, employed, *n* (%)	299 (34)	259 (18)	275 (32)	282 (18)
Missing	8 (1)	17 (1)	10 (1)	18 (1)
Type of work, *n* (%)				
Physical	50 (17)	55 (21)	67 (24)	73 (26)
Mental	105 (25)	90 (35)	83 (30)	67 (24)
Both	122 (41)	107 (41)	115 (42)	139 (49)
Missing	22 (7)	7 (3)	10 (4)	3 (1)
Living alone, yes, *n* (%)	96 (11)	406 (30)	85 (10)	428 (28)
Missing	44 (5)	97 (7)	53 (6)	105 (7)
Preoperative HOOS/KOOS Score, mean (SD)**				
Pain	43 (18)	36 (18)	43 (17)	37 (17)
Symptoms	44 (19)	38 (18)	54 (18)	48 (18)
Daily living	45 (19)	38 (19)	50 (19)	43 (18)
QoL	20 (20)	16 (19)	29 (16)	25 (15)
Sports	31 (17)	27 (16)	15 (17)	8 (13)
SF-12, mean (SD)				
PCS	33 (10)	31 (9)	34 (9)	31 (9)
Missing, *n* (%)	67 (7)	151 (11)	57 (60)	170 (11)
MCS	55 (10)	53 (11)	56 (9)	54 (10)
Missing, *n* (%)	67 (7)	151 (11)	57 (6)	170 (11)
Preoperative expectations, mean (SD)	82 (19)	78 (19)	72 (18)	69 (18)
Missing, *n* (%)	30 (3)	77 (5)	10 (1)	26 (2)

*THA* total hip arthroplasty, *TKA* total knee arthroplasty, *HOOS* Hip Disability and Osteoarthritis Outcome Score, *KOOS* Knee Injury and Osteoarthritis Outcome Score, *SF-12* Short Form-12 survey, *PCS* component score for physical health score, *MCS* component score for mental health score

**HOOS/KOOS scores are complete for approximately 75% of patients, as it was replaced with the HOOS-PS/KOOS-PS after January 2017

### Comparisons of preoperative expectations

The proportion of patients scoring individual items as applicable varied considerably. Expectations re*lated to work, sexual activity, psychological well-being and no need for a cane, crutch or walker* were relatively less often considered as “applicable” (Supplementary Tables 2-A and 2-B) in both hip and knee populations. As for THA patients, men and women showed a differences in proportions of ≥ 5% in “applicability” on four items, with only the item *eliminate the need for pain relief medication* higher among women (Supplementary Table 2-A). Nevertheless, only the items *sexual activity* and *return to work* showed a difference in the proportion of ≥ 10% in reporting an item as “applicable”, in favor of men. 9/20 items showed a difference between men and women regarding applicability. Overall, we found differences of ≥ 10% (OR ≥ 1.1) in the probability of having higher preoperative expectations, in favor of men in 16/20 HSS-HRES items (Table 2)*.*

**Table 2:** Total hip arthroplasty (THA): preoperative expectations using the HSS-HRES

	Applicableǂ		Back to normal (%)		Much improved (%)		Moderately improved (%)		Slightly improved (%)		OR [95% CI]*
	♂, *n*	♀, *n*	♂	♀	♂	♀	♂	♀	♂	♀	
Relief of pain during the day	749 (98)	1230 (99)	65	64	31	31	3	4	1	1	1.1 [0.9–1.4]
Relief of pain during sleeping	807 (93)	1304 (96)	74	69	21	27	3	4	2	1	1.3 [1.0–1.5]
Improve walking ability											
Short distances (in house)	829 (98)	1262 (98)	73	70	23	26	3	4	2	1	1.2 [1.0–1.5]
Middle-long distances (< 1.5 km)	826 (97)	1252 (97)	52	59	33	32	13	7	3	2	1.2 [1.0–1.4]
Long distances (> 1.5 km)	808 (94)	1206 (93)	52	49	33	33	13	13	3	6	1.2 [1.0–1.4]
No need for cane, crutch or walker	624 (72)	987 (71)	88	84	8	9	3	4	1	2	1.4 [1.0–1.8]
Ability to stand better	833 (95)	1320 (94)	78	75	18	20	3	5	1	1	1.2 [0.9–1.4]
Getting rid of limp	813 (92)	1258 (90)	77	77	21	18	2	4	1	1	1.0 [0.8–1.2]
Walking stairs	837 (95)	1318 (94)	73	70	23	23	4	5	1	2	1.2 [1.0–1.4]
Getting out of bed, chair or car	851 (97)	1388 (99)	72	71	23	24	3	5	1	1	1.1 [0.9–1.3]
Eliminate need for pain relief medication	662 (75)	1175 (84)	87	81	10	14	2	4	2	1	1.5 [1.1–1.9]
Be able to put on shoes and socks	797 (90)	1236 (88)	81	80	15	15	2	4	2	2	1.0 [0.8–1.3]
Be able to do paid work	319 (37)	282 (21)	88	89	9	7	2	3	2	2	1.1 [0.7–1.7]
Join recreational activities (dancing, going out on trips)	703 (80)	1087 (78)	73	70	21	23	4	6	2	1	1.2 [1.0–1.5]
Improve ability to perform daily activities in and around the house	828 (94)	1346 (96)	73	71	22	21	3	6	1	2	1.2 [1.0–1.5]
Improve ability to do sports	749 (85)	1116 (84)	56	59	36	31	6	9	2	2	0.9 [0.8–1.1]
Ability to cut toenails	773 (88)	1190 (85)	67	69	24	22	6	6	3	3	1.0 [0.8–1.2]
Social life	746 (85)	1128 (81)	76	71	18	21	6	6	1	2	1.4 [1.1–1.6]
Sexual activity	640 (73)	820 (59)	79	76	13	16	5	6	3	1	1.2 [0.9–1.5]
Psychological well-being	610 (70)	853 (61)	83	78	11	14	4	5	2	3	1.5 [1.1–1.9]

*HSS-HRES* Hospital for Special Surgery Hip Replacement Expectations Survey, ♂ men, ♀ women

ǂPreoperative expectations proportions based on patients that reported preoperative items as “applicable”; more information on preoperative “not applicable”/“applicable” population in Supplementary Table 2-A; *comparison of preoperative expectations in men and women by means of ordinal logistic regression (presented as odds ratio (OR) with corresponding 95% confidence interval (95% CI); men as reference category); with OR > 1 indicating lower preoperative expectations among women as a higher HSS-HRES score indicates a lower expectation

In the TKA population, men and women showed a difference in the proportions of ≥ 5% in five items regarding “applicable” (Supplementary Table 2-B). Similar to the THA population, expectations of *sexual activity* and *return to work* showed differences in proportions of ≥ 10% in favor of men. On 9/19 items, applicability was different between men and women. Overall, we found differences of ≥ 10% (OR ≥ 1.1) in the probability of having higher preoperative expectations, in favor of men in 14/19 HSS-KRES items (Table 3). In general, men had higher expectations compared to women.

**Table 3:** Total knee arthroplasty (TKA): preoperative expectations using the HSS-KRES

	Applicableǂ		Back to normal (%)		Much improved (%)		Moderately improved (%)		Slightly improved (%)		OR [95% CI]*
	♂, *n*	♀, *n*	♂	♀	♂	♀	♂	♀	♂	♀	
Relief of pain during the day	823 (99)	1479 (99)	49	50	46	44	4	6	1	1	1.0 [0.9–1.2]
Improve walking ability											
Short distances (in house)	740 (98)	1320 (99)	58	58	37	35	3	6	2	1	1.0 [0.9–1.2]
Middle-long distances (< 1.5 km)	746 (98)	1318 (97)	48	44	42	42	9	12	2	2	1.2 [1.0–1.4]
Long distances (> 1.5 km)	764 (96)	1272 (92)	38	37	45	43	12	15	5	6	1.2 [1.0–1.4]
No need for cane, crutch or walker	530 (65)	1036 (70)	86	83	10	12	2	4	1	2	1.1 [0.9–1.5]
Be able to stretch the knee	773 (93)	1426 (95)	65	62	30	30	4	7	2	1	1.1 [1.1–1.3]
Improve walking upstairs	810 (97)	1465 (97)	59	56	34	34	5	9	2	2	1.2 [1.0–1.4]
Improve walking downstairs	808 (97)	1459 (97)	58	56	36	34	5	8	1	2	1.1 [1.0–1.4]
Being able to kneel down	794 (96)	1390 (92)	31	30	46	37	16	19	7	15	1.3 [1.1–1.6]
Being able to squat	791 (96)	1388 (93)	29	28	45	37	19	21	7	15	1.3 [1.1–1.6]
Being able to travel by public transportation (bus, tram or train)	569 (69)	1128 (75)	74	67	21	22	4	8	1	3	1.4 [1.1–1.7]
Be able to do paid work	287 (36)	342 (24)	79	81	15	14	2	3	3	2	0.8 [0.6–1.2]
Join recreational activities (dancing, going out on trips)	688 (83)	1185 (79)	57	55	33	33	8	8	2	3	1.0 [0.9–1.3]
Improve ability to perform daily activities in and around the house	743 (89)	1361 (90)	74	71	19	21	5	7	2	2	1.2 [1.0–1.4]
Improve ability to do sports	720 (86)	1223 (81)	40	42	44	41	13	14	2	4	1.0 [0.8–1.1]
Being able to change positions (getting up, sitting down)	797 (95)	1450 (96)	59	57	34	33	5	9	2	2	1.1 [0.9–1.3]
Social life	679 (82)	1212 (80)	61	58	32	31	6	8	1	3	1.2 [1.0–1.4]
Sexual activity	557 (68)	751 (51)	72	68	17	20	7	9	4	3	1.2 [0.9–1.5]
Psychological well-being	516 (62)	872 (58)	73	69	17	20	7	7	4	5	1.3 [1.0–1.6]

*HSS-KRES* Hospital for Special Surgery Knee Replacement Expectations Survey, ♂ men, ♀ women

ǂPreoperative expectations proportions based on patients that reported preoperative items as “applicable”; more information on “not applicable”/“applicable” population in Supplementary Table 2-B; *comparison of preoperative expectations in men and women by means ordinal logistic regression (presented as odds ratio (OR) with corresponding 95% confidence interval (95% CI); men as reference category); with OR > 1 indicating lower preoperative expectations among women, as a higher HSS-KRES score indicates a lower expectation

### Fulfillment of expectations

Tables 4 and 5 show an overview of the applicability and fulfillment of expectations 1 year after surgery*.* In the THA population, 7/20 HSS-HRES items showed ≥ 5% difference in the proportions of “applicability” between men and women, of which 6 items had higher proportions among men than women (Table 4). Similar to the preoperative results, the item *ability to do work* showed a difference in prevalence of ≥ 10% in favor of men (83% vs 70%). 12/20 items showed a difference between men and women regarding applicability. All items were fulfilled in ≥ 60% of men, and on 18/20 HSS-HRES items, ≥ 60% of women reported fulfilled expectations. The following expectations were most often fulfilled: *be able to do paid work* (men: 91%; women: 88%) and *no need for a cane, crutch or walker* (men: 85%; women: 78%). Although the majority of all patients had fulfilled their expectations on the HSS-HRES items, the proportion of men with fulfilled expectations was higher on all items. 18/20 HSS-HRES items showed a difference in proportions of ≥ 5%, in favor of men, in the proportion of fulfilled expectations, and ≥ 10% differences in *walking stairs* and *sexual activity* (Table 4).

**Table 4:** Total hip arthroplasty (THA): fulfillment of preoperative expectations on HSS-HRES 1 year after surgery

			Applicableǂ		95% CI*	Fulfilled		95% CI*
	♂, *n*	♀, *n*	♂, *n* (%)	♀, *n* (%)		♂, %	♀, %	
Relief of pain during the day	579	907	552 (95)	860 (95)	0.69–1.49	74	68	0.60–0.96
Relief of pain during sleeping	594	903	559 (94)	853 (95)	0.97–1.70	76	70	0.57–0.92
Improve walking ability								
Short distances (in house)	645	928	629 (98)	891 (96)	0.50–1.13	77	71	0.55–0.88
Middle-long distances (< 1.5 km)	638	901	604 (95)	806 (90)	0.40–0.75	72	65	0.60–0.94
Long distances (> 1.5 km)	624	872	558 (89)	731 (84)	0.50–0.80	68	61	0.58–0.93
No need for cane, crutch or walker	477	717	338 (68)	488 (68)	0.75–1.08	85	78	0.43–0.88
Ability to stand better	648	983	617 (95)	892 (93)	0.59–1.05	76	68	0.53–0.83
Getting rid of limp	629	937	577 (92)	921 (87)	0.43–0.77	74	68	0.43–0.67
Walking stairs	655	982	627 (96)	814 (91)	0.52–0.83	72	59	0.61–0.97
Getting out of bed, chair or car	660	1044	640 (97)	985 (94)	0.69–1.41	68	62	0.63–0.96
Eliminate need for pain relief medication	513	880	313 (61)	591 (67)	1.22–1.77	84	76	0.42–0.86
Be able to put on shoes and socks	621	931	585 (94)	866 (93)	0.59–0.95	71	67	0.67–1.06
Be able to do paid work	319	283	264 (83)	199 (70)	0.30–0.46	91	88	0.42–1.38
Join recreational activities (dancing, going out on trips)	556	827	451 (81)	605 (73)	0.56–0.82	74	69	0.58–1.00
Improve ability to perform daily activities in and around the house	652	1021	610 (94)	929 (91)	0.69–1.19	72	63	0.53–0.82
Improve ability to do sports	589	847	490 (83)	666 (79)	0.56–0.82	68	64	0.67–1.09
Ability to cut toenails	600	910	532 (89)	750 (82)	0.52–0.79	64	55	0.55–0.86
Social life	599	850	517 (86)	701 (83)	0.55–0.82	79	72	0.53–0.91
Sexual activity	504	631	403 (80)	477 (76)	0.45–0.64	83	72	0.38–0.73
Psychological well-being	477	637	417 (87)	524 (83)	0.51–0.73	83	75	0.43–0.83

*HSS-HRES* Hospital for Special Surgery Hip Replacement Expectations Survey, ♂ men, ♀ women

ǂFulfillment of preoperative expectations proportions based on patients that reported both preoperative and postoperative items as “applicable”; *comparison of fulfillment of expectations in men and women by means of Chi-square test, with corresponding 95% confidence interval (CI)

**Table 5:** Total knee arthroplasty (TKA): fulfillment of preoperative expectations on HSS-KRES 1 year after surgery

			Applicableǂ		95% CI*	Fulfilled		95% CI*
	♂, *n*	♀, *n*	♂, *n* (%)	♀, *n* (%)		♂, %	♀, %	
Relief of pain during the day	629	1102	603 (96)	1053 (96)	0.72–1.55	67	65	0.72–1.10
Improve walking ability								
Short distances (in house)	560	925	545 (97)	896 (97)	0.80–1.75	73	66	0.59–0.94
Middle–long distances (< 1.5 km)	564	930	531 (94)	843 (91)	0.49–0.91	67	63	0.66–1.04
Long distances (> 1.5 km)	579	910	518 (90)	771 (85)	0.43–0.70	66	61	0.65–1.03
No need for cane, crutch or walker	377	750	220 (58)	461 (62)	1.04–1.54	85	80	0.45–1.06
Be able to stretch the knee	598	1070	573 (94)	1015 (95)	0.86–1.48	75	77	0.91–1.46
Improve walking upstairs	624	1106	597 (96)	1040 (94)	0.60–1.14	66	58	0.57–0.87
Improve walking downstairs	629	1101	601 (96)	1025 (93)	0.56–1.04	62	56	0.62–0.94
Being able to kneel down	607	1024	524 (86)	760 (74)	0.37–0.58	49	42	0.60–0.94
Being able to squat	611	1033	526 (86)	792 (77)	0.42–0.66	54	49	0.65–1.01
Being able to travel by public transportation (bus, tram or train)	429	834	346 (81)	631 (76)	0.90–1.30	80	76	0.57–1.08
Be able to do paid work	287	342	224 (78)	244 (71)	0.42–0.64	85	78	0.68–1.93
Join recreational activities (dancing, going out on trips)	529	879	436 (82)	669 (76)	0.57–0.84	76	63	0.42–0.72
Improve ability to perform daily activities in and around the house	573	1009	539 (94)	965 (96)	0.87–1.42	78	73	0.59–0.97
Improve ability to do sports	559	933	442 (79)	702 (75)	0.60–0.88	67	65	0.70–1.15
Being able to change positions (getting up, sitting down)	616	1103	581 (94)	1038 (94)	0.76–1.35	65	64	0.78–1.19
Social life	522	919	449 (86)	757 (84)	0.68–1.01	73	68	0.63–1.06
Sexual activity	429	570	330 (77)	419 (74)	0.43–0.62	76	77	0.75–1.49
Psychological well-being	401	646	337 (84)	510 (79)	0.61–0.88	80	77	0.62–1.22

*HSS-KRES* Hospital for Special Surgery Knee Replacement Expectations Survey, ♂ men, ♀ women

ǂFulfillment of preoperative expectations proportions based on patients that reported both preoperative and postoperative items as “applicable”; *comparison of fulfillment of expectations in men and women by means of Chi-square test, with corresponding 95% confidence interval (CI)

In the TKA population, 7/19 HSS-KRES items showed ≥ 5% difference in proportion “applicable” when comparing men and women, with higher proportion of “applicable” rates among men, which included mainly functionally related expectations (Table 4). On 10/19 items, applicability was different between men and women. The majority of items of the HSS-KRES had a proportion of ≥ 60% with fulfilled expectations: 17/19 items among men and 15/19 among women. In accordance with the THA population, the items *be able to do paid work* (men: 85%; women: 78%) and no *need for a cane, crutch or walker* (men: 85%; women: 80%) were most often fulfilled expectations. Overall, men had a larger proportions of fulfilled expectations on 15 items than women. Additionally we found ≥ 5% difference, in favor of men, in the proportion of fulfilled expectations on ten items between men and women, which were mainly functionally related (Table 5).

## Discussion

The most important finding of this study was that men and women have different perceptions of preoperative expectations regarding outcome of THA or TKA. One year after THA or TKA, they differ in their fulfillment of expectations. More items are perceived as applicable to men than women, in particular in terms of sexual activity and ability to work (difference in prevalence > 10%). Other items showed only small differences in the score “applicable”. Men reported higher preoperative expectations related to the ability to perform functional activities compared to women, and men more often fulfilled their preoperative expectations 1 year after THA and TKA than women.

As previously reported, men more often report that the item regarding *sexual activity* applies to them [15]. In addition, men more often report that the item *being able to do paid work* applied to them. In our population, women more often live alone and are less often employed, which could partly explain these differences. Elderly people who live alone more often tend to report a lower frequency of sexual activity and consider it less important than people living with a spouse [26]. In addition, men and women have different preoperative expectations on several items of the HSS-HRES and HSS-KRES. Men are more likely to expect to return to normal in terms of pain relief and the ability to perform basic functional activities, while women expect moderate to much improvement in these items. The latter is in accordance with the results of other studies in OA patients after a THA or TKA [11, 12]. Some authors suggest that these differences in preoperative expectations could be related to the fact that women are more likely to opt for OA treatment at a later stage of the disease, possibly because they are more afraid of TKA/THA surgery and suffer from OA pain for longer than men [7].

Our findings on the fulfillment of expectations in terms of sex are in line with other studies [16, 17]. The high proportion of fulfilled expectations on the item *no need for a cane, crutch or walker* could be explained by the improved functional status and walking ability in many patients after THA and TKA [27]. Furthermore, many patients are fulfilled with the expectation *be able to do paid work.* This can be explained by the previously established association between patients’ beliefs and preoperative expectations and return to work after surgery [28]. With regard to unfulfilled expectations, we find that the expectation of *the ability to cut toenails* is often unfulfilled after THA, with percentages ranging from 64 to 55% in men and women. Items with a high proportion of unfulfilled expectations among TKA patients were: *the ability to kneel or squat,* and *walking up and downstairs* [16]. Studies addressing the effect of sex on fulfillment of expectations after THA or TKA are scarce. Previous studies [17, 29] show that women less often experience fulfillment in expectations 1 year postoperatively, but failed to include the specific items in which differences are present. Furthermore, unfulfilled expectations are known to be a principal source of patient dissatisfaction [30]. In addition to this finding, other specialties within medicine have identified sex as an independent predictor for unsatisfactory outcomes 1 year after surgery, such as patients with sciatica [31].

Our findings are in accordance with previous studies that suggest women have a worse preoperative disease state. For instance they score worse on function and pain [3]. Differences between preferences and expectations prior to THA and TKA could be possible explanations for the difference in disease state preoperatively. Lower expectations of surgical interventions, such as THA or TKA, can lead to postponing surgery. Contrary to this, others suggest that worse preoperative disease state can lead to lower preoperative expectations and explain a difference between men and women [32].

Based on our findings, we suggest to take these sex specific differences into account when informing patients in a shared decision-making process for THA or TKA. Also future research should focus on the underlying reasons that could explain the differences found in this study, such as differences in life situations which could influence needs and demands. Patient-specific education provides more realistic information about expectations of outcomes, which could lead to better postoperative outcomes, patient satisfaction, and increased fulfillment of preoperative expectations [33]. Hence, it has also been shown that sex disparities in postoperative TKA expectations can be targeted with a decision aid [34].

Despite the prospective nature of this study for both preoperative and postoperative fulfillment scores, there are some limitations. First, for the assessment of expectations and fulfillment we used standardized expectation surveys, which do not include the patient’s own individual expectations regarding other activities or aspects of life. Second, with regard to the option “not applicable” of the HSS-HRES/HSS-KRES, patients might have different reasons to score “not applicable”. Possible explanations can be: unable to respond, not doing a certain activity or having expectations that were lower than the available scoring options. We are not able to specify these different reasons. Nevertheless, this study showed that men reported the item on sexual activity and ability to do paid work more often as “applicable”, and were more often employed and less often lived alone, compared to women. This supports our reasoning that reporting an item as “not applicable”, is identical to ‘not doing’ the activity, and is less related to the severity of the hip/knee complaints. Although the questionnaires were specifically developed for THA/TKA outcomes, the meaning of “back to normal” could have been interpreted differently by patients, as no detailed description of this response option was provided (i.e., before OA-related symptoms started, or before THA/TKA). Furthermore, not being able to indicate expectations such as ‘no improvement’ or ‘worsening’, although not a desirable outcome of elective surgery, could have resulted in an overestimation in preoperative expectations and an underestimation in fulfillment. We did not include ‘exceeded’ as a separate category, as there is a potential ceiling effect in this questionnaire when calculating exceeded expectations: if a patient preoperatively expects an item to go back to normal, this patient will not have the ability to exceed his/her expectations after surgery, as there is no category above ‘back to normal’. As the proportion of patients reporting an item as ‘back to normal’ before surgery is large (> 50% on almost all items), we categorized exceeded expectations alongside fulfilled expectations.

Men had higher expectations and more often fulfilled their expectations 1 year after THA and TKA surgery compared to women. Men’s expectations were mainly related to the ability to perform functional activities, while women were more concerned with the performance of activities of daily living. A deeper understanding of the impact of sex on expectations, both before and after THA/TKA, helps informing patients and the shared decision-making process. As a result, orthopedic surgeons and other health-care providers are able to more specifically target expectations in both men and women and to provide a more tailored expectation management, which finally could diminish sex disparities. Our recommendation for future research is to evaluate whether such tailored shared decision-making process, in which the specific expectations of men and women are included, indeed diminishes differences in fulfillment of expectations, thereby aiming at an optimal balance between preoperative expectations and fulfilled expectations after a THA/TKA. In addition, future research should focus on the underlying reasons of the sex differences found.

## Supplementary Information

Below is the link to the electronic supplementary material.

Supplementary file1 (DOCX 250 KB)

## Data Availability

The data that support the findings of this study are available from the corresponding author, D.A.J.M. Latijnhouwers, upon reasonable request.

## Code availability

The code used to analyze the current study is available from the corresponding author on reasonable request.
