# Supplementary material for: Sex differences in perceived expectations of the outcome of total hip and knee arthroplasties and their fulfillment: an observational cohort study
Source: Rheumatol Int. 2022 Nov 28;43(5):911–22. doi: 10.1007/s00296-022-05240-y (PMC10073060; doi:10.1007/s00296-022-05240-y)
Supplement: Supplementary file 1 — Supplementary file1 (DOCX 250 KB) [file 296_2022_5240_MOESM1_ESM.docx]

| **Supplementary table 1-A. Preoperative characteristics of THA and TKA patients with and without response to the preoperative expectations questionnaire** | | | | | |
| --- | --- | --- | --- | --- | --- |
|  | | **THA population** | | **TKA population** | |
|  | | **With**  **(n = 2333)** | **Without**  **(n = 237)** | **With**  **(n = 2398)** | **Without**  **(n = 194)** |
| Age (years), mean (SD) | | 69 (9) | 71 (9) | 68 (9) | 70 (9) |
| BMI, mean (SD) | | 27 (4) | 28 (4) | 30 (5) | 30 (5) |
| Smoking, yes, n (%) | | 136 (6) | 18 (8) | 131 (6) | 17 (9) |
| Comorbidities (%) | Non-musculoskeletal | 549 (24) | 14 (7) | 447 (34) | 13 (7) |
|  | Musculoskeletal | 534 (23) | 23 (10) | 283 (12) | 14 (7) |
|  | Both | 525 (23) | 7 (6) | 824 (27) | 21 (11) |
|  | None  Missing | 476 (20)  249 (11) | 17 (3)  176 (74) | 568 (19)  276 (12) | 13 (7)  133 (69) |
| Work status, employed, n (%)  Missing | | 558 (24)  25 (1) | 5 (2)  176 (74) | 557 (23)  28 (1) | 13 (7)  131 (68) |
| Living alone, yes, n (%)  Missing | | 502 (23)  141 (6) | 16 (7)  163 (69) | 513 (21)  158 (7) | 19 (10)  123 (63) |
| Preoperative HOOS/KOOS, mean (SD)** | Pain | 38 (19) | 46 (22) | 39 (18) | 36 (18) |
|  | Symptoms | 41 (19) | 47 (25) | 50 (18) | 49 (16) |
|  | Daily Living | 41 (19) | 47 (20) | 45 (19) | 40 (21) |
|  | QoL | 29 (17) | 40 (23) | 26 (16) | 27 (18) |
|  | Sports | 18 (19) | 13 (20) | 10 (15) | 10 (17) |
| SF-12, mean (SD) | PCS | 32 (9) | 32 (10) | 32 (9) | 34 (11) |
|  | MCS | 54 (10) | 48 (11) | 55 (10) | 50 (12) |
| **Legend to Supplementary Table 1-A:**  **: HOOS/KOOS scores are complete for approximately 75% of patients, as it was replaced with the HOOS-PS/KOOS-PS after January 2017Note: THA: total hip arthroplasty; TKA: total knee arthroplasty; HOOS: Hip Disability and Osteoarthritis Outcome Score; KOOS: Knee Injury and Osteoarthritis Outcome Score; SF-12: Short form-12 survey; PCS: component score for physical health score; MCS: component score for mental health score. | | | | | |

| **Supplementary table 1-B. Preoperative characteristics of THA and TKA patients with and without follow-up** | | | | | |
| --- | --- | --- | --- | --- | --- |
|  | | **THA population** | | **TKA population** | |
|  | | **Without**  **(n = 2333)** | **With**  **(n = 1878)** | **Without**  **(n = 2398)** | **With**  **(n = 1887)** |
| Age (years), mean (SD) | | 69 (9) | 69 (9) | 68 (9) | 68 (8) |
| BMI, mean (SD) | | 27 (4) | 27 (4) | 30 (5) | 29 (5) |
| Smoking, yes, n (%) | | 136 (6) | 93 (5) | 131 (6) | 95 (5) |
| Comorbidities (%) | Non-musculoskeletal | 549 (24) | 458 (24) | 447 (34) | 633 (34) |
|  | Musculoskeletal | 534 (23) | 414 (22) | 283 (12) | 219 (12) |
|  | Both | 525 (23) | 398 (21) | 824 (27) | 433 (23) |
|  | None  Missing | 476 (20)  249 (11) | 385 (21)  224 (12) | 568 (19)  276 (12) | 346 (18)  257 (14) |
| Work status, employed, n (%)  Missing | | 558 (24)  25 (1) | 437 (23)  15 (2) | 557 (23)  28 (1) | 426 (23)  21 (2) |
| Living alone, yes, n (%)  Missing | | 502 (23)  141 (6) | 378 (20)  135 (7) | 513 (21)  158 (7) | 384 (20)  162 (9) |
| Preoperative HOOS/KOOS, mean (SD)* | Pain | 38 (19) | 39 (19) | 39 (18) | 39 (17) |
|  | Symptoms | 41 (19) | 41 (19) | 50 (18) | 50 (18) |
|  | Daily Living | 41 (19) | 41 (19) | 45 (19) | 46 (18) |
|  | QoL | 29 (17) | 30 (17) | 26 (16) | 27 (15) |
|  | Sports | 18 (19) | 18 (19) | 10 (15) | 11 (15) |
| SF-12, mean (SD) | PCS | 32 (9) | 32 (9) | 32 (9) | 32 (9) |
|  | MCS | 54 (10) | 54 (10) | 55 (10) | 55 (10) |
| **Legend to Supplementary Table 1-B:**  *: HOOS/KOOS scores are complete for approximately 75% of patients, as it was replaced with the HOOS-PS/KOOS-PS after January 2017Note: THA: total hip arthroplasty; TKA: total knee arthroplasty; HOOS: Hip Disability and Osteoarthritis Outcome Score; KOOS: Knee Injury and Osteoarthritis Outcome Score; SF-12: Short form-12 survey; PCS: component score for physical health score; MCS: component score for mental health score. | | | | | |

| **Supplementary table 2-A. Total Hip Arthroplasty (THA): Preoperative Expectations using the HSS-HRES** | | | | | |
| --- | --- | --- | --- | --- | --- |
|  |  | | **Applicable**ǂ | | **95% CI*** |
|  | Men, n (%) | Women, n (%) | Men, n (%) | Women, n (%) |  |
| *Relief of pain during the day* | 763 | 1,240 | 749 (98) | 1,230 (99) | 0.17 – 0.15 |
| *Relief of pain during sleeping* | 868 | 1,364 | 807 (93) | 1,304 (96) | **0.39 – 0.87** |
| Improve walking ability | | | | | |
| *Short distances (in house)* | 843 | 1,286 | 829 (98) | 1,262 (98) | 0.50 – 2.86 |
| *Middle-long distances (<1.5 km)* | 848 | 1,294 | 826 (97) | 1,252 (97) | 0.73 – 2.55 |
| *Long distances (>1.5 km)* | 856 | 1,301 | 808 (94) | 1,206 (93) | 0.92 – 2.17 |
|  |  |  |  |  |  |
| *No need for cane, crutch or walker* | 872 | 1,382 | 624 (72) | 987 (71) | 0.82 – 1.25 |
| *Ability to stand better* | 880 | 1,399 | 833 (95) | 1,320 (94) | 0.62 – 1.42 |
| *Getting rid of limp* | 881 | 1,393 | 813 (92) | 1,258 (90) | 0.74 – 1.78 |
| *Walking stairs* | 879 | 1,397 | 837 (95) | 1,318 (94) | 0.88 – 1.76 |
| *Getting out of bed, chair or car* | 880 | 1,406 | 851 (97) | 1,388 (99) | **0.16 – 0.64** |
| *Eliminate need for pain relief medication* | 881 | 1,400 | 662 (75) | 1,175 (84) | **0.44 – 0.70** |
| *Be able to put on shoes and socks* | 884 | 1,405 | 797 (90) | 1,236 (88) | 0.82 – 1.50 |
| *Be able to do paid work* | 867 | 1,348 | 319 (37) | 282 (21) | **1.86 – 2.87** |
| *Join recreational activities (dancing, going out on trips)* | 874 | 1,392 | 703 (80) | 1,087 (78) | 0.90 – 1.46 |
| *Improve ability to perform daily activities in and around the house* | 878 | 1,406 | 828 (94) | 1,346 (96) | 0.41 – 1.03 |
| *Improve ability to do sports* | 880 | 1,391 | 749 (85) | 1,116 (84) | **1.04 – 1.75** |
| *Ability to cut toenails* | 880 | 1,404 | 773 (88) | 1,190 (85) | 0.85 – 1.49 |
| *Social life* | 879 | 1,394 | 746 (85) | 1,128 (81) | **1.18 - .99** |
| *Sexual activity* | 875 | 1,387 | 640 (73) | 820 (59) | **1.47 – 2.23** |
| *Psychological well-being* | 877 | 1,398 | 610 (70) | 853 (61) | **1.19 – 1.79** |
| **Legend to Supplementary table 2-A:**  ǂ Preoperative expectations proportions based on applicable population  *Comparison of preoperative expectations in men and women by means of Chi-square test, with corresponding 95% Confidence Interval (CI)  Note: HSS-HRES: Hospital For Special Surgery Hip Replacement Expectations Survey; | | | | | |

| **Supplementary table 2-B. Total Knee Arthroplasty (TKA): Preoperative Expectations using the HSS-KRES** | | | | | |
| --- | --- | --- | --- | --- | --- |
|  |  | | **Applicable** | | **95%CI*** |
|  | Men, n | Women, n | Men, n (%) | Women, n (%) |  |
| *Relief of pain during the day* | 830 | 1,490 | 823 (99) | 1,479 (99) | 0.18 – 1.76 |
| Improve walking ability | | | | | |
| *Short distances (in house)* | 756 | 1,335 | 740 (98) | 1,320 (99) | 0.26 – 1.32 |
| *Middle-long distances (<1.5 km)* | 760 | 1,360 | 746 (98) | 1,318 (97) | 0.75 – 2.92 |
| *Long distances (>1.5 km)* | 796 | 1,385 | 764 (96) | 1,272 (92) | **1.26 – 3.16** |
|  |  |  |  |  |  |
| *No need for cane, crutch or walker* | 822 | 1,481 | 530 (65) | 1,036 (70) | **0.58 – 0.88** |
| *Be able to stretch the knee* | 834 | 1,509 | 773 (93) | 1,426 (95) | **0.47 – 1.00** |
| *Improve walking upstairs* | 835 | 1,514 | 810 (97) | 1,465 (97) | 0.49 – 1.45 |
| *Improve walking downstairs* | 832 | 1,506 | 808 (97) | 1,459 (97) | 0.44 – 1.33 |
| *Being able to kneel down* | 831 | 1,506 | 794 (96) | 1,390 (92) | **1.04 – 2.46** |
| *Being able to squat* | 828 | 1,500 | 791 (96) | 1,388 (93) | 0.89 – 2.08 |
| *Being able to travel by public transportation (bus, tram or train)* | 824 | 1,497 | 569 (69) | 1,128 (75) | **0.57 – 0.87** |
| *Be able to do paid work* | 801 | 1,405 | 287 (36) | 342 (24) | **1.31 – 2.03** |
| *Join recreational activities (dancing, going out on trips)* | 834 | 1,500 | 688 (83) | 1,185 (79) | **0.95 – 1.56** |
| *Improve ability to perform daily activities in and around the house* | 831 | 1,512 | 743 (89) | 1,361 (90) | 0.68 – 1.25 |
| *Improve ability to do sports* | 836 | 1,502 | 720 (86) | 1,223 (81) | 1.00 – 1.71 |
| *Being able to change positions (getting up, sitting down)* | 835 | 1,511 | 797 (95) | 1,450 (96) | 0.43 – 1.07 |
| *Social life* | 832 | 1,508 | 679 (82) | 1,212 (80) | 0.77 – 1.26 |
| *Sexual activity* | 822 | 1,482 | 557 (68) | 751 (51) | **1.57 – 2.35** |
| *Psychological well-being* | 828 | 1,501 | 516 (62) | 872 (58) | **1.05 – 1.56** |
| **Legend to Supplementary table 2-B:**  ǂ Preoperative expectations proportions based on applicable population  * Comparison of preoperative expectations in men and women by means of Chi-square test, with corresponding 95% Confidence Interval (CI)  Note: HSS-KRES: Hospital For Special Surgery Knee Replacement Expectations Survey; | | | | | |

**Supplement A. Expectations questionnaire**The versions of the HSS-HRES and HSS-KRES developed by van den Akker-Scheek et al. (van den Akker-Scheek, I., van Raay, J. J., Reininga, I. H., Bulstra, S. K., Zijlstra, W., & Stevens, M. (2010). Reliability and concurrent validity of the Dutch hip and knee replacement expectations surveys. BMC musculoskeletal disorders, 11(1), 1-8.) were used in this study. The developer of the questionnaires was informed and gave consent to a Dutch translation of the Expectations Surveys (Carol Mancuso, MD, Hospital for Special Surgery, personal communication, 2008). The studies of Mancuso et al. (mentioned below) include a detailed description of the English version of the questionnaires.

References for English versions:

*Mancuso CA, Wentzel CH, Ghomrawi HMK, Kelly BT. Hip Preservation Surgery Expectations Survey: A New Method to Measure Patients' Preoperative Expectations. Arthroscopy 2017; 33: 959-968.*

*Mancuso CA, Sculco TP, Wickiewicz TL, Jones EC, Robbins L, Warren RF, et al. Patients' expectations of knee surgery. JBJS 2001; 83: 1005-1012.*

Reference for Dutch version:

van den Akker-Scheek I, van Raay JJ, Reininga IH, Bulstra SK, Zijlstra W, Stevens M. Reliability and concurrent validity of the Dutch hip and knee replacement expectations surveys. BMC Musculoskelet Disord 2010; 11: 242

**Validated Dutch of the Hospital for Special Surgery Hip Replacement Expectations Survey**


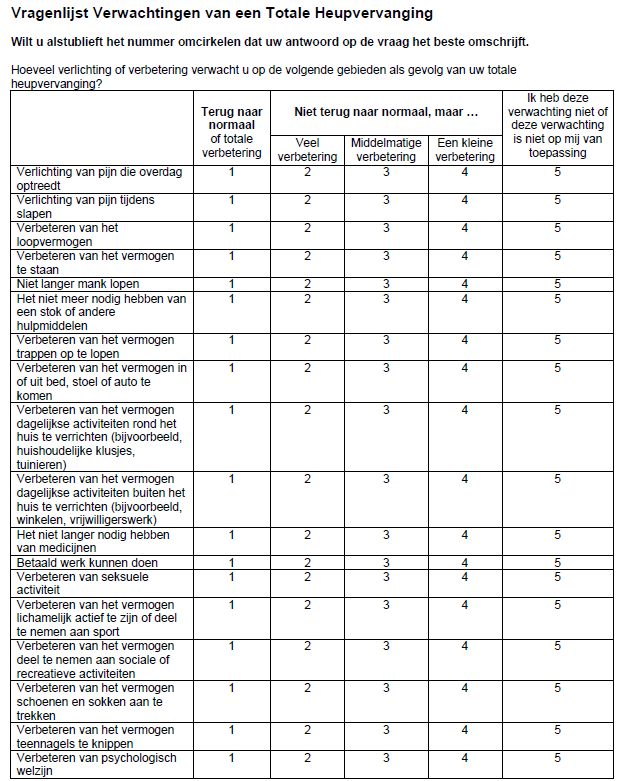


*

*: subdivided in the following subquestions: i) korte afstanden (binnenshuis, een huizenblok), ii) middellange afstanden (een stukje lopen, tot 1,5 km), iii) lange afstanden (meer dan 1,5 km)

**Validated Dutch of the Hospital for Special Surgery Knee Replacement Expectations Survey**


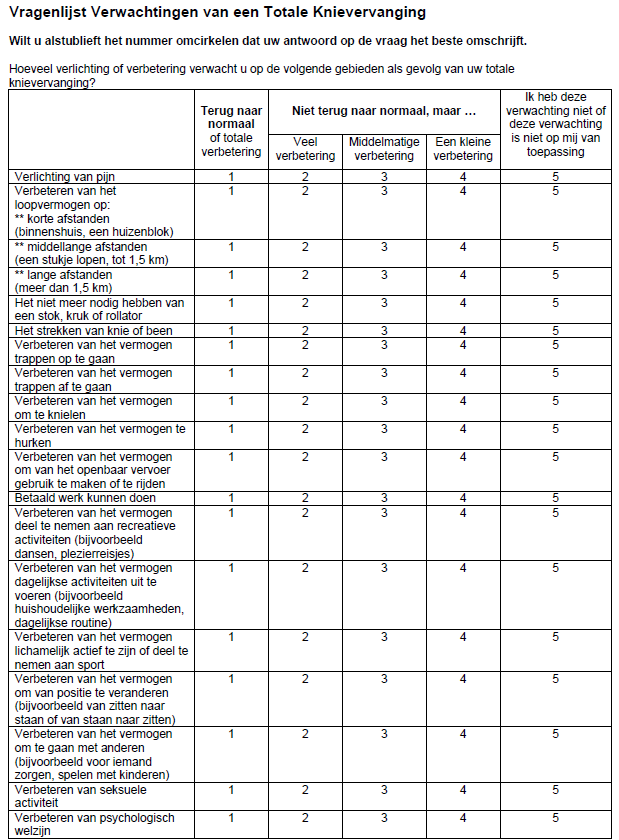


**Supplement B. Explanation regarding our unadjusted analysis approach:**

Hernán (2008) provided the following definitions for a confounding variable: “can be used to block a backdoor path between exposure and outcome”, “any variable that can be used to reduce [confounding] bias” and “any variable that is necessary to eliminate the bias in the analysis” *(Hernán MA. Confounding. In: Everitt B, Melnick E, editors. Encyclopedia of Quantitative Risk Assessment and Analysis. John Wiley & Sons Chichester, United Kingdom: 2008. pp. 353–362.)*

Below we have created a Directed Acyclic Graph (DAG) to explain why we have not adjusted for additional covariates (*Supplementary figure 1*). DAGs represent causal relationships among variables, and can be used to determine the variables on which it is necessary to condition to control for confounding in the estimation of causal effects.


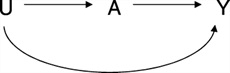


*Supplementary figure 1*. Example illustrating confounding by health status. Y indicates disease; A, exposure; U, health status

*(VanderWeele, Tyler J.a; Hernán, Miguel A.b; Robins, James M.b,c Causal Directed Acyclic Graphs and the Direction of Unmeasured Confounding Bias, Epidemiology: September 2008 - Volume 19 - Issue 5 - p 720-728 doi: 10.1097/EDE.0b013e3181810e29)*

We investigated if sex affects expectations, indicated by the arrow between “Sex (male/female)”/exposure and “Expectations”/outcome (or dependent variable) in *Supplementary figure 2*. Indeed there could be other factors, alongside sex, that affect expectations, such as living situation and having paid work. However, these factors are a result of sex and are therefore not a “cause” of sex. Hence, being female could affect your living situation and having paid work, and therefore indirectly via living situation/paid work affect expectations. Nevertheless, as made visible using the DAG below, these ‘covariates’ are within the causal pathway between “sex” and “expectations”, or so-called ‘mediators’. Therefore, adjusting for these covariates/mediators would lead to non-preferable adjustments within the causal pathway. To our knowledge there are no covariates ‘causing’ sex, that could affect expectations.


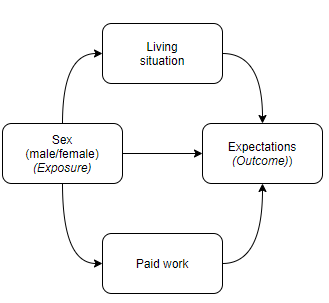


Supplementary figure 2: Directed Acyclic Graph
